# Identifying Phosphodiesterase‐5 Inhibitors with Drug Repurposing Approach: Implications in Vasodysfunctional Disorders

**DOI:** 10.1002/open.202300196

**Published:** 2023-12-07

**Authors:** Mohd Shahnawaz Khan, Hamza Ahmad Mohammad, Moyad Shahwan, Dharmendra Kumar Yadav, Saleha Anwar, Anas Shamsi

**Affiliations:** ^1^ Department of Biochemistry College of Science King Saud University, KSA; ^2^ Center for Medical and Bio-Allied Health Sciences Research Ajman University (UAE); ^3^ Gachon Institute of Pharmaceutical Science and Department of Pharmacy College of Pharmacy Gachon University Incheon (Republic of Korea; ^4^ Centre for Interdisciplinary Research in Basic Sciences Jamia Millia Islamia, Jamia Nagar New Delhi 110025 India

**Keywords:** drug repurposing, drug design, molecular dynamics, signaling molecules, virtual screening

## Abstract

Phosphodiesterase type 5 (PDE5) is a multidomain protein that plays a crucial role in regulating cellular cyclic guanosine monophosphate (cGMP), a key signaling molecule involved in various physiological processes. Dysregulation of PDE5 and cGMP signaling is associated with a range of vasodysfunctional disorders, necessitating the development of effective therapeutic interventions. This study adopts comprehensive approach, combining virtual screening and molecular dynamics (MD) simulations, to repurpose FDA‐approved drugs as potential PDE5 inhibitors. The initial focus involves selecting compounds based on their binding affinity. Shortlisted compounds undergo a meticulous analysis for their drug profiling and biological significance, followed by the activity evaluation and interaction analysis. Notably, based on binding potential and drug profiling, two molecules, Dutasteride and Spironolactone, demonstrate strong potential as PDE5 inhibitors. Furthermore, all atom MD simulations were employed (500 ns) to explore dynamic behavior of Dutasteride and Spironolactone in complexes with PDE5. Principal components analysis (PCA) and free energy landscape (FEL) analyses are further leveraged to decipher that the binding of Dutasteride and Spironolactone stabilizes the structure of PDE5 with minimal conformational changes. In summary, Dutasteride and Spironolactone exhibit remarkable affinity for PDE5 and possess characteristics that suggest their potential as therapeutic agents for conditions associated with PDE5 dysfunction.

## Graphical abstract

**Phosphodiesterase type 5** plays a crucial role in regulating key signaling molecules involved in various physiological processes. This study combines virtual screening and molecular dynamics simulations to investigate the repurposing of FDA‐approved drugs as potential PDE5 inhibitors.

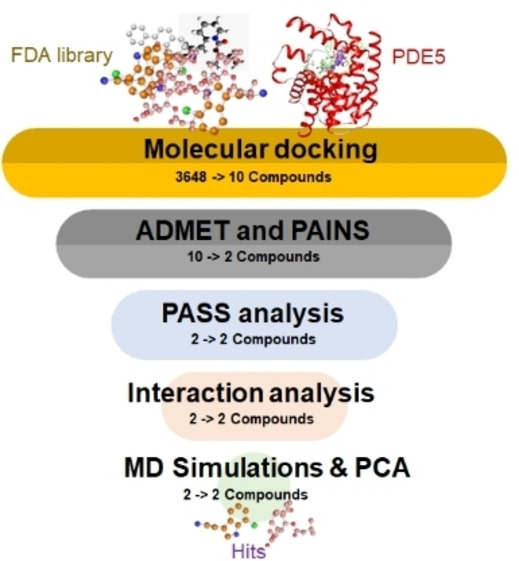

## Introduction

Phosphodiesterase type 5 (PDE5) stands as a pivotal enzyme in the regulation of cyclic guanosine monophosphate (cGMP) levels within cells, exerting a profound influence on various physiological processes.^[1]^ The molecule cGMP holds a prominent role in intracellular signaling cascades, predominantly recognized for its involvement in smooth muscle relaxation and vasodilation of blood vessels.^[2]^ These actions are particularly significant within the cardiovascular system, where balanced vasodilation is critical in maintaining optimal blood pressure and circulation.^[3]^ Given its central role in vascular function, PDE5 has garnered substantial attention as a promising target for therapeutic intervention.^[4]^ Vasodilation, orchestrated by cGMP, not only facilitates efficient blood flow but also plays a key role in conditions associated with vascular dysfunction.^[5]^ Pathological conditions such as pulmonary arterial hypertension (PAH) and benign prostatic hyperplasia (BPH) underscore the intricate interplay of vasodilation and smooth muscle relaxation.^[6]^ Moreover, Raynaud's phenomenon, a vascular disorder causing extremity discoloration due to vasoconstriction, and broader cardiovascular health concerns are also linked to cGMP imbalance.^[7]^

PDE5 is positioned at the intersection of these critical physiological processes and has gained prominence as a therapeutic target for drug development.^[8]^ The central role of PDE5 in regulating cGMP levels positions it as an influential modulator of vasodilation and smooth muscle relaxation.^[9]^ Initial exploration of PDE5 inhibitors primarily focused on addressing erectile dysfunction. The discovery and clinical success of Sildenafil, the first FDA‐approved PDE5 inhibitor, revolutionized the field and demonstrated the therapeutic potential of targeting this enzyme.^[10]^ However, the expanding understanding of PDE5′s functions has prompted researchers to explore its role beyond erectile dysfunction, propelling investigations into repurposing PDE5 inhibitors for broader applications.^[11]^ The multifaceted potential of PDE5 inhibition extends beyond erectile dysfunction to encompass a spectrum of vasodysfunctional disorders.^[12]^ This is underscored by the clinical application of PDE5 inhibitors, such as tadalafil, vardenafil, and avanafil, in diverse contexts.^[13]^ The concept of drug repurposing, wherein existing drugs are explored for new therapeutic indications, provides a promising avenue for expediting the development of treatments for various conditions.^[14]^ This approach capitalizes on existing safety and efficacy data, potentially shortening the drug development timeline and reducing costs.^[15]^ Consequently, there is a growing interest in utilizing repurposing strategies to identify novel applications for PDE5 inhibitors beyond their original indications.

This study presents a comprehensive approach that capitalizes on computational methodologies to identify potential PDE5 inhibitors from the existing pool of FDA‐approved drugs. Virtual screening is a powerful in silico technique that enables the rapid evaluation of compound libraries against target proteins.^[16]^ Combining this with molecular dynamics (MD) simulations, which provide insights into the dynamic behavior of protein‐ligand interactions over time, offers a robust platform for predicting and understanding binding interactions.^[17]^ Our aim was to systematically explore the repurposing potential of FDA‐approved drugs as inhibitors of PDE5. To achieve this, we initiated our approach with molecular docking, prioritizing the assessment of binding affinity scores to pinpoint compounds with a strong likelihood of interacting with PDE5. After pinpointing high‐affinity candidates, we proceeded with comprehensive validation through specialized evaluations, including assessments such as the prediction of activity spectra for substances (PASS).^[18]^ Subsequently, shortlisted compounds underwent interaction analysis to refine the selection and enhance the assessment of their potency as PDE5 inhibitors.

Among the identified hit molecules, two candidates, Dutasteride and Spironolactone, displayed significant affinity and specificity for the PDE5 binding site. To delve deeper into their binding dynamics and potential inhibitory effects, we performed extensive MD simulations. These simulations provided insights into the dynamic behavior of Dutasteride and Spironolactone in complexes with PDE5, shedding light on their interactions, stability, and impact on PDE5 conformation. In summary, our study presents a systematic exploration of repurposing FDA‐approved drugs as potential PDE5 inhibitors through an integrated computational pipeline. The identified drugs, Dutasteride and Spironolactone, emerged as strong contenders with notable affinity and characteristics that indicate their potential as therapeutic agents for conditions linked to PDE5 dysfunction. Integrating virtual screening and MD simulations offers a comprehensive platform for identifying and understanding potential drug interactions, contributing to the acceleration of drug repurposing efforts. Overall, this study underscores the broader potential of PDE5 inhibition and showcases the efficiency of computational approaches in the realm of drug discovery and repurposing.

## Experimental Section

### Dataset Preparation

The crystal structure of human PDE5 was obtained from the RCSB Protein Data Bank (PDB accession code: 3HC8).^[19]^ Prior to the computational analysis, the protein structure was prepared using PyMOL,^[20]^ and Swiss‐PDB‐Viewer.^[21]^ The residues were renumbered as per the UniProt sequence entry (UniProt accession code: O76074). Water molecules, heteroatoms and the co‐crystallized ligand were removed, and hydrogen atoms were added using PyMOL molecular visualization software. The protein was then energy‐minimized in Swiss‐PDB‐Viewer using GROMOS43B1 force field to relax its structure while avoiding steric clashes. A diverse set of 3648 FDA‐approved drug molecules was compiled from the publicly available DrugBank database.^[22]^ This library was curated to encompass a broad spectrum of chemical structures and therapeutic indications.

### Molecular Docking

Molecular docking‐based screening plays a crucial role in drug discovery and development by computationally evaluating a vast collection of small molecules against specific protein targets.^[23]^ This process helps narrow down the extensive list of potential therapeutic candidates.^[16]^ In this study, a compound library was meticulously prepared and subjected to docking screening against the binding site of PDE5 using the AutoDock Vina software.^[24]^ For the molecular docking‐based virtual screening, grid dimensions were set as 74 Å for X, 76 Å for Y, and 66 Å for Z coordinates. The central location was defined with X at 9.36 Å, Y at −0.089 Å, and Z at 19.559 Å. A consistent grid spacing of 1 Å was maintained, and exhaustiveness was set to 8. These parameters were carefully adjusted to ensure a comprehensive exploration of ligand conformations and orientations, aiming to cover a wide range of potential binding poses. Following the screening process, compounds were ranked based on their binding affinity scores. The top‐ranking compounds were then chosen for further analysis of drug profiling and PASS prediction.^[18]^

### Compound Selection and PASS Evaluation

To ensure the reliability of the top‐ranking hits, the selected compounds were subjected to explore their drug profiles and biological significance, followed by PASS evaluation.^[18]^ The PASS predictions estimated the likelihood of the compounds having specific biological activities based on their chemical structures. In the PASS analysis, the ‘Pa’ and ‘Pi’ are descriptors used to represent the predicted biological activity and inactivity probabilities, respectively, for a given chemical compound in various predefined activity categories. These descriptors provide information about the likelihood of a compound exhibiting a specific biological activity or inactivity. This analysis provides valuable information regarding the compound's biological activities, enabling a comprehensive assessment of their diverse pharmacological profiles. This approach capitalizes on chemical‐biological interactions to uncover the biological attributes of the chemicals being studied. To execute this analysis, the PASS server was employed to dissect the biological traits of the compounds that were identified from the previous filter.

### Interaction Analysis

Compounds selected based on their biological properties underwent a comprehensive interaction analysis using PyMOL^[20]^ and Discovery Studio Visualizer.^[25]^ This evaluation played a pivotal role in refining the compound selection process, focusing on their ability to interact favorably with crucial residues within the PDE5 binding site. The docked conformations of the selected compounds were subjected to an in‐depth analysis to explore their potential interactions with PDE5. This analysis encompassed hydrogen bonding, π‐π interactions, hydrophobic interactions, and other relevant interactions to elucidate binding modes and identify the key residues involved in ligand binding. Based on this thorough interaction analysis, the conformations that exhibited specific interactions with PDE5 binding and active‐site residues were retained for further consideration and exploration in all‐atom MD simulations.

### MD Simulations

The chosen compounds underwent all‐atom MD simulations using GROMACS^[26]^ to evaluate their stability and binding dynamics over 500 ns time. The MD simulations commenced with the PDE5 structure and its complexes with the selected ligands obtained through molecular docking. Ligand topologies were prepared using the ATB server.^[27]^ To establish solvated systems, the water molecules around protein‐ligand complexes were added through the TIP3P water model. Counterions (Na^+^ or Cl^−^) were introduced for system neutrality. Energy minimization was initiated on the solvated system, utilizing the steepest descent energy minimization algorithm to mitigate steric clashes and optimize geometry. Equilibration ensued, gradually heating the system from 0 K to 300 K over 100 ps with a thermostat. During this process, position restraints on protein and ligand‐heavy atoms facilitated solvent relaxation. The GROMOS 54 A7 force field was employed for position restraints and MD simulation initialization.^[28]^ Periodic boundary conditions and the particle mesh Ewald (PME) method managed long‐range electrostatic interactions. A 2‐fs time step was utilized, ensuring constant temperature and pressure. The LINCS algorithm maintained bond length constraints, enabling a longer time step. Post‐simulation trajectories were analyzed using various utilities of the GROMACS package. Dynamic parameters such as root mean square deviation (RMSD) and root mean square fluctuation (RMSF) gauged structural stability and detected flexible regions across the simulation. Other metrics like radius of gyration (*R*g), solvent accessibility surface area (SASA), hydrogen bonds (H‐bonds), and secondary structure dynamics were computed and visualized.

### Principal Component Analysis

PCA is a compelling methodology to dissect the collective movements and dynamics of proteins and protein‐ligand complexes.^[29]^ By discovering the significant motions and correlations between atoms or atom groups, PCA simplifies intricate MD trajectory data. This technique is instrumental in unveiling the fundamental movements that navigate the system‘s conformational changes and dynamics. From the MD trajectory involving PDE5 and its bound complexes with Dutasteride and Spironolactone along with the reference inhibitor, Sildenafil, a matrix encompassing atomic coordinates was generated. Subsequently, the covariance matrix emerged from this coordinate matrix, with its components quantifying correlations between atom pairs. Utilizing eigenvector‐eigenvalue decomposition, the covariance matrix was diagonalized. In this context, the eigenvectors represent the principal components (PCs), with their associated eigenvalues indicating the extent of variance elucidated by each PC. By projecting the MD trajectory onto the PCs, particularly PC1 and PC2, a reduced‐dimensional representation of the data was constructed. This briefly captures the essential dynamics of all the systems. PCA employs the calculation and diagonalization of the covariance matrix which can be calculated utilizing the following formula:
(1)

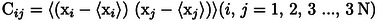




Where, x_*i*_ = x_*j*_ is the coordinate of the *i* ^th^/*j* ^th^ atom of the system, while <–> denotes the ensemble average.

### Free Energy Landscape

FEL analysis is a technique used to visualize the thermodynamic stability and conformational states of a molecular system.^[30]^ In the context of protein‐ligand interactions, FEL analysis helps understand the stability of ligand binding and the various conformations the complex can adopt.^[31]^ FEL analysis was employed to map out energetically favorable conformations within the PDE5‐ligand complexes. This methodology allowed for identifying stable binding modes and provided insights into the complex interplay between ligand binding and dynamic structural shifts within the system. Through FEL analysis, different binding poses and their corresponding energetic states were revealed, offering a holistic perspective on the binding mechanism and its intricate connection to the dynamic characteristics of the protein‐ligand complexes. The FELs can be constructed utilizing the following formula:−
(2)

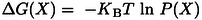




Where *K*_B_ and *T* are the Boltzmann constant and absolute temperature, respectively. Δ*G*(*X*) is the probability distribution of the molecular system along with the principal components.

## Results and Discussion

### Molecular Docking Screening

A thorough molecular docking‐based virtual screening assessed which and how well FDA‐approved compounds interacted with PDE5. This critical stage aimed to identify potential molecules that strongly bind to PDE5. By analyzing docking scores, the compounds were systematically ranked, and from this ranking, the highest‐scoring compounds were carefully selected for a more detailed examination. This selection process was mainly driven by the goal of pinpointing ligands with exceptionally strong binding to PDE5. The selected compounds demonstrated significant affinity for PDE5′s binding site, as evident from the docking results provided in Table 1. Remarkably, out of the extensive pool of 3648 compounds initially considered, the top 10 compounds emerged as promising candidates, showcasing binding affinity scores ranging from −10.7 kcal/mol to −11.7 kcal/mol. All these selected hits showed higher affinity than the reference inhibitor, Sildenafil. These findings offer insights into the remarkable binding strength of these chosen compounds toward PDE5. The results highlight the potential of these compounds as robust candidates to advance into subsequent stages of drug development. Their robust binding affinity suggests their promise for further exploration and potential utilization in harnessing therapeutic benefits in PDE5‐associated diseases.

**Table 1:** The top 10 hits and their docking score with PDE5.

S. No.	Molecule	Binding Free Energy [kcal/mol]	pKi	Ligand Efficiency [kcal/mol/ non‐H atom]	Torsional Energy
1.	Paritaprevir	−11.7	8.58	0.2127	2.1791
2.	Dutasteride	−11.5	8.43	0.3108	1.2452
3.	Bisdequalinium Chloride	−11.4	8.36	0.2591	0
4.	Midostaurin	−11.4	8.36	0.2651	1.8678
5.	Algestone Acetophenide	−11.3	8.29	0.3424	0.6226
6.	Fendosal	−11.1	8.14	0.3828	1.5565
7.	Pyronaridine	−10.9	7.99	0.2946	2.4904
8.	Ergotamine	−10.8	7.92	0.2512	1.5565
9.	Benzquercin	−10.8	7.92	0.1895	4.9808
10.	Spironolactone	−10.7	7.85	0.369	0.6226
11.	Sildenafil	−8.2	6.01	0.2485	2.1791

### PASS Evaluation

The highest‐ranking compounds from the docking screening underwent a thorough analysis for their drug profiling and biological relevance, followed by PASS evaluation. This step ensured that the chosen compounds possessed appropriate chemical attributes and anticipated biological activities relevant to inhibiting PDE5. The PASS analysis was meticulously carried out to shed light on the potential pharmacological capabilities of these compounds. The PASS assessment revealed a wide range of possible pharmacological activities linked to Dutasteride and Spironolactone. The broad scope of projected activities suggested their adaptability and capacity to influence different biological pathways, offering promise across diverse medical contexts. The insights gleaned from the PASS analysis played a pivotal role in exploring the potential attributes of these clarified hit compounds. Summarizing the outcomes, Table 2 summarizes the compounds, their inherent biological characteristics, and the confidence level in these predictions. The findings conclusively indicated that Dutasteride and Spironolactone showcased significant attributes as an androgen antagonist, prostate disorders and PBH treatments, androgen antagonist, antihypertensive, and cerebrovascular disorders treatment. These predictions were further substantiated by impressive Pa values ranging from 0,356 to 0,988. Of particular importance, as highlighted by the comprehensive PASS analysis, Dutasteride and Spironolactone emerged as promising contenders for inclusion in PDE5‐associated therapeutic pursuits.

**Table 2:** PASS properties of the selected molecules and their scores. The ‘Pa’ and ‘Pi’ represent the probabilities to be active and inactive, respectively, for a given chemical compound in various activity categories.

S. No.	Molecule	Pa	Pi	Biological Activity
1.	Dutasteride	0,988	0,002	Androgen antagonist
		0,909	0,003	Prostate disorders treatment
		0,893	0,002	Prostatic (benign) hyperplasia treatment
		0,852	0,001	5‐Alpha‐reductase inhibitor
		0,356	0,077	Menopausal disorders treatment
2.	Spironolactone	0,991	0,001	Diuretic
		0,958	0,002	Androgen antagonist
		0,804	0,005	Antihypertensive
		0,719	0,005	Prostate disorders treatment
		0,598	0,003	Cerebrovascular disorders treatment
3.	Sildenafil	0,811	0,002	Phosphodiesterase V inhibitor
		0,867	0,003	Male reproductive disfunction treatment
		0,771	0,002	Cyclic GMP phosphodi‐ esterase inhibitor
		0,288	0,082	Antihypertensive
		0,215	0,152	Cardiotonic

### Interaction Analysis

**Figure 1:**
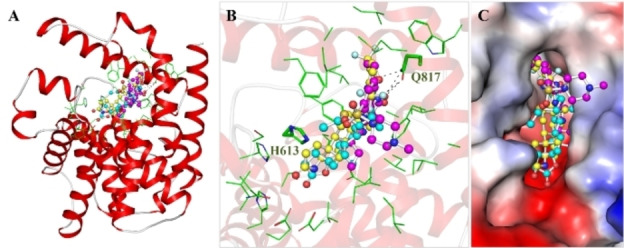
Intermolecular interactions of Dutasteride (yellow), Spironolactone (cyan), and Sildenafil (magenta) with PDE5. (A) Cartoon, (B) Magnified cartoon, and (C) Surface view of PDE5 with Dutasteride, Spironolactone, and Sildenafil.

The compounds with favorable drug profiling and biological properties and appreciable docking scores, were subjected to in‐depth analysis for their interactions with crucial residues located in the PDE5 binding site. This evaluation encompassed the study of hydrogen bonding patterns, hydrophobic interactions, and other bonding modes to determine the binding quality. This step aimed to enhance the evaluation of their efficacy as PDE5 inhibitors. Through this analysis, significant interactions emerged, including hydrogen bonding, π‐π stacking, and hydrophobic interactions, which unfolded between the compounds and essential amino acid residues. These interactions provided invaluable insights into the stabilizing forces that underlie the binding of Dutasteride and Spironolactone to PDE5 (Figure 1A). Upon thorough examination, the interactions exhibited notable engagement with key PDE5 PDEase domain residues, with particular emphasis on Gln817 and His613, among others (Figure 1B). This analysis accentuated the noteworthy interactions, especially the direct contact established with His613–the active site residue (proton donor) of PDE5 (http://www.uniprot.org/uniprotkb/O76083/entry). Both compounds showed close interactions with Gln817, the 3′,5′‐cyclic GMP binding site of PDE5.^[32]^ At the same time, both compounds share similar interactions with several common residues as the reference inhibitor, Sildenafil.^[32]^ Importantly, the binding pocket of PDE5 showcased compatibility with these compounds, further underscoring their potential as promising candidates (Figure 1C). Previous studies have consistently showcased that the interaction pattern displayed by small molecule inhibitors with PDE5 results in substantially inhibiting its function.[^19^, ^32^] This inhibitory effect holds promising potential for therapeutic applications, providing valuable therapeutic advantages.

**Figure 2:**
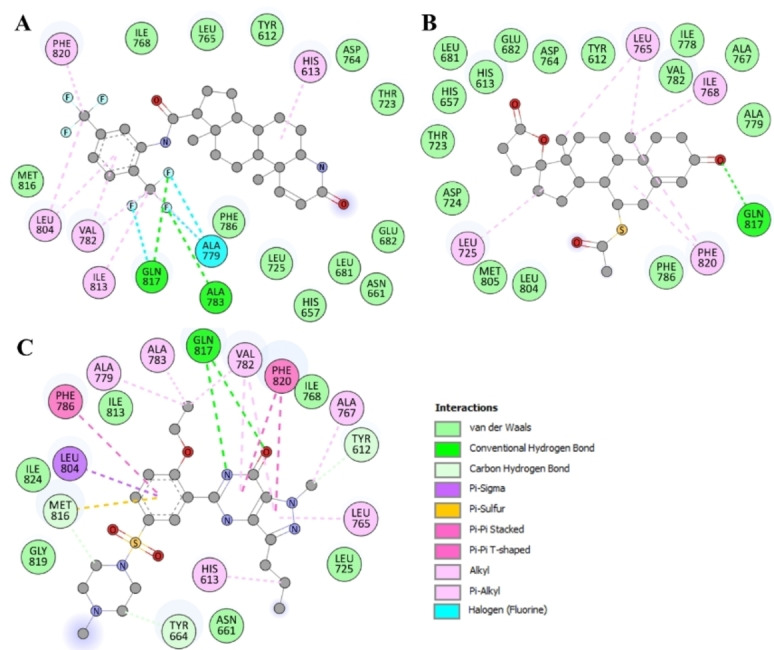
Representation of intermolecular interactions showing detailed interactions of (**A**) Dutasteride, (**B**) Spironolactone, and (**C**) Sildenafil with PDE5.

Further exploration revealed a noteworthy revelation: both compounds effectively occupied the active site and cGMP binding pocket within PDE5, engaging numerous vital residues of the enzyme (Figure 2). In the case of the PDE5‐Dutasteride complex, the binding was fortified by the establishment of two H‐bonds involving Ala783 and Gln817, along with three halogen bonds with Gln817 and Ala779 and seven alkyl bonds with five different residues. Furthermore, a significant number of Van der Waals contacts contributed to the interaction (Figure 2A). Likewise, in the PDE5‐Spironolactone complex, the stability was upheld by interactions such as a hydrogen bond with Gln817 and six alkyl bonds with four different amino acid residues (Figure 2B). Additionally, the PDE5‐Spironolactone complex benefited from several Van der Waals contacts, further bolstering its stability. Additionally, both compounds displayed notable interactions with Gln817 through hydrogen bonding and share several common interactions with the reference inhibitor, Sildenafil (Figure 2C). These intricate interaction patterns shed light on the intricate interplay between the ligands and the delicate residues within the PDE5 binding site. Previous research has consistently demonstrated that small molecule inhibitors interact with PDE5 in a manner that significantly inhibits its function.^[19]^ Overall, the interaction analysis elucidated the mechanism by which these compounds establish their binding affinity and potentially influence the biological activity of PDE5. However, experimental data would provide more robust and direct evidence of the binding and inhibition of PDE5 by these compounds.

### MD Simulations

#### PDE5 Structural Dynamics and Compactness

**Figure 3:**
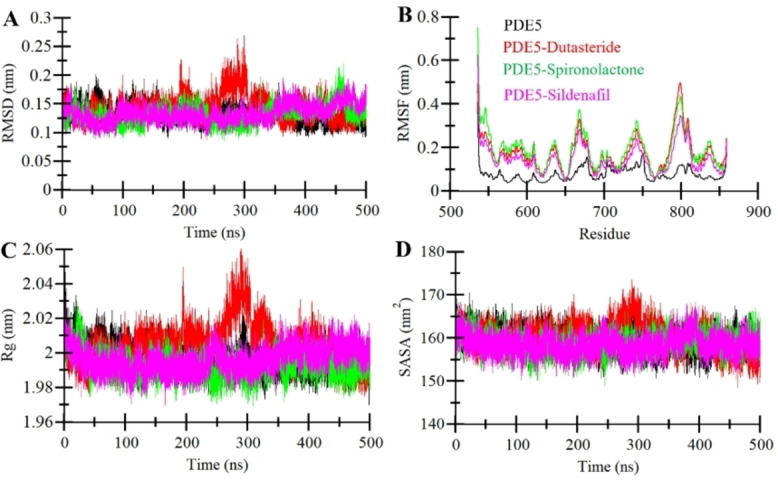
Structural dynamics of PDE5 (black), PDE5‐Dutasteride (red), PDE5‐Spironolactone (green), and PDE5‐Silenafil (magenta) (**A**) RMSD, (**B**) RMSF, (**C**) *R*g, and (**D**) SASA calculated after 500 ns of MD trajectories.

The dynamic behaviors of each complex were comprehensively explored through an analysis of key properties, including RMSD and RMSF, throughout the simulation. This thorough investigation aimed to reveal any potential structural variations in PDE5 and its associated docked complexes. This is crucial because while docking studies offer a snapshot of static protein‐ligand interactions, MD simulations enable the observation of the dynamic interplay of these interactions over time. Figure 3A visually portrays the fluctuations in RMSD values for each system over the 500 ns MD trajectories. All complexes demonstrated remarkable stability, with no significant deviations observed during the simulation. Notably, the dynamic equilibrium between the complexes and PDE5 remained largely constant, underscoring the robustness of these interactions in a dynamic context. To quantitatively estimate the dynamic stability of the complexes, the RMSD values of the backbone carbon atoms were examined. This approach succinctly reflects the structural integrity of the initial complex configurations. Monitoring the fluctuations in these RMSD values offers valuable insights into the strength of the complexes within the dynamic simulation environment. The consistent maintenance of low RMSD values further reinforces the stability and viability of Dutasteride and Spironolactone as effective PDE5 inhibitors.

To examine the stability of residues within the binding pocket throughout MD simulations, we thoroughly assessed and presented the RMSF profiles of all PDE5 residues. The computation of RMSF for each residue provided insights into the extent of fluctuations experienced by individual amino acids within the protein during the entire 500 ns MD simulation period. This analysis enabled us to evaluate the potential impact of ligand binding on the dynamic structure of the binding pocket. During the RMSF calculation, we determined the average fluctuation for each PDE5 residue before and after the binding of the compounds (Figure 3B). Importantly, a noticeable pattern seen in the RMSF profiles highlighted that the residues in proximity to the binding pocket exhibited notably lower levels of fluctuation compared to residues in other parts of the protein structure. This observation signifies the inherent stability maintained by the binding pocket over time. Moreover, the binding‐induced stabilization effect emphasizes the role of Dutasteride and Spironolactone, similar to the reference molecule Sildenafil, in enhancing the structural integrity of the binding site. The diminished fluctuations observed in the binding pocket residues validate the compounds′ ability to engage consistently and robustly with the protein. This, in turn, contributes to the overall stability of the PDE5‐ligand complexes throughout the entirety of the simulation duration (Figure 3B).

The analysis of *R*g serves as an invaluable tool for investigating the compactness and folding mechanisms inherent to proteins, including their interactions with ligands within protein‐ligand complexes.^[33]^ The *R*g metric offers insights into the packing density of a protein; higher *R*g values indicate a looser packing, while lower values suggest tighter packing. Within the context of MD experiments, the *R*g analysis allows us to observe how ligand molecules impact the conformational dynamics of proteins. Here, we employed *R*g analysis to delve into the influence of the interactions between PDE5 and its respective ligands on the protein‘s conformational arrangement. Figure 3C visually represents the *R*g analysis, displaying the fluctuations in *R*g values for each complex over the MD simulations. Notably, when PDE5 formed a complex with Spironolactone, it exhibited the lowest *R*g value throughout the simulation as compared to other systems. Overall, the finding indicates that the presence of the ligands leads to a compact structural configuration for PDE5 without any major fluctuations and unfolding events. Noteworthy is the observation that ligand binding seems to encourage a conformational structure that maintains the protein‘s compactness while elevating the overall stability of the PDE5‐ligand complexes.

Furthermore, we investigated the evolution of SASA values throughout the simulation to uncover the folding and unfolding behaviors of PDE5, both before and after interactions with the ligands. SASA values provide insight into the extent to which individual amino acids within a protein are exposed to the surrounding solvent environment. This information is visually depicted in Figure 3D. Interestingly, the SASA values align harmoniously with the observed trends in *R*g across the entire 500 ns simulation trajectories. A slight reduction in SASA values following ligand binding signifies a tighter packing of the protein, particularly pronounced in the case of Spironolactone and Sildenafil. This suggests an improved stability and compactness of the docked complexes during the simulation period.

#### Dynamics of Hydrogen Bonds

**Figure 4:**
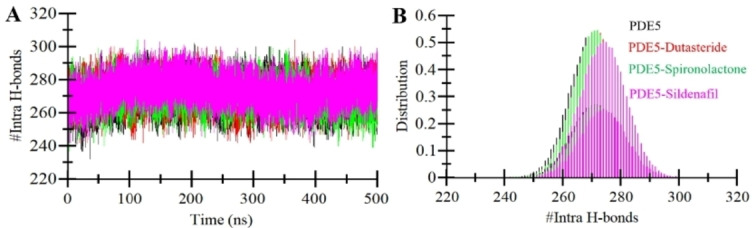
The time evolution of H‐bonds in PDE5. (**A**) Intramolecular and (**B**) Intermolecular hydrogen bond analysis in PDE5 (black), PDE5‐Dutasteride (red), PDE5‐Spironolactone (green), and PDE5‐Sildenafil (magenta).

Maintaining the structural stability of proteins hinges on establishing intramolecular H‐bonds, crucial interactions that contribute to upholding the native conformation of the protein.^[34]^ The intricate dynamics of intramolecular H‐bonds can be explained through the MD simulations, which offer insights into ligand binding dynamics in protein structure. In this study, our primary objective centered around examining intramolecular H‐bonds and elucidating their dynamic changes throughout MD simulations to unveil the structural consequences of ligand binding. We employed a meticulous approach by establishing a distance threshold of 3.5 Å, allowing us to systematically monitor the formation and evolution of intramolecular H‐bonds within PDE5, both in the absence and presence of ligand binding (Figure 4A). Furthermore, our analysis of the probability distribution function (PDF) demonstrated that the distribution of these bonds exhibited significant overlap in values between PDE5 configurations before and after ligand binding (Figure 4B). Overall, the study suggests that PDE5 maintains the overall distribution of the intramolecular H‐bonds network and, thus, overall structural integrity.

**Figure 5:**
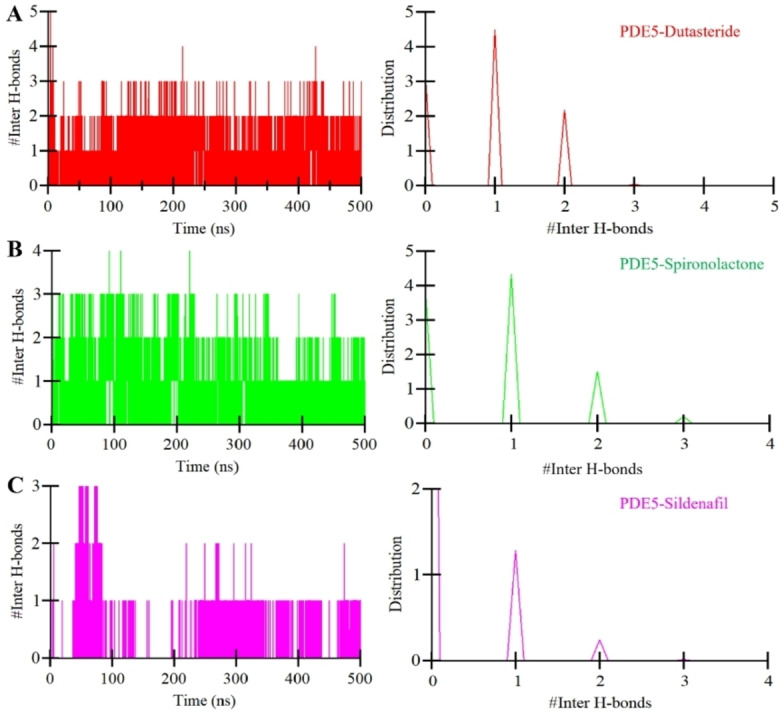
The time evolution of Intermolecular hydrogen bond analysis in PDE5 (black), PDE5‐Dutasteride (red), PDE5‐Spironolactone (green), and PDE5‐Sildenafil (magenta).

Further, to evaluate the complex integrity, we also calculated the intermolecular H‐bonds form between PDE5 and the selected ligands. Figure 5 visually represents the outcomes of intermolecular H‐bond analysis across all three systems examined. Figure 5A vividly portrays the landscape of H‐bonds formed within the PDE5‐Dutasteride complex. Here, we observed the formation of an average of 2 H‐bonds, occasionally increasing up to 4–5. At the same time, the PDE5‐Spironolactone complex also exhibited an average of 2 H‐bonds, occasionally reaching 3–4 bonds (Figure 5B). Both compounds showed higher intermolecular H‐bond stability than the reference molecule, Sildenafil, which showed only 1 H‐bond, occasionally increasing to 3 (Figure 5C). The analysis of the PDF plots revealed a persistent pattern in the distribution of intermolecular H‐bonds across all three complexes (Figure 5, right panel**s**). Specifically, a prevalent observation was the frequent occurrence of a single H‐bond in each complex. Importantly, this study provided evidence that the positions of the compounds remained stable and closely aligned with their initial docking configurations, indicating the presence of a robust and reliable binding mode.

#### Secondary Structure Dynamics

**Figure 6:**
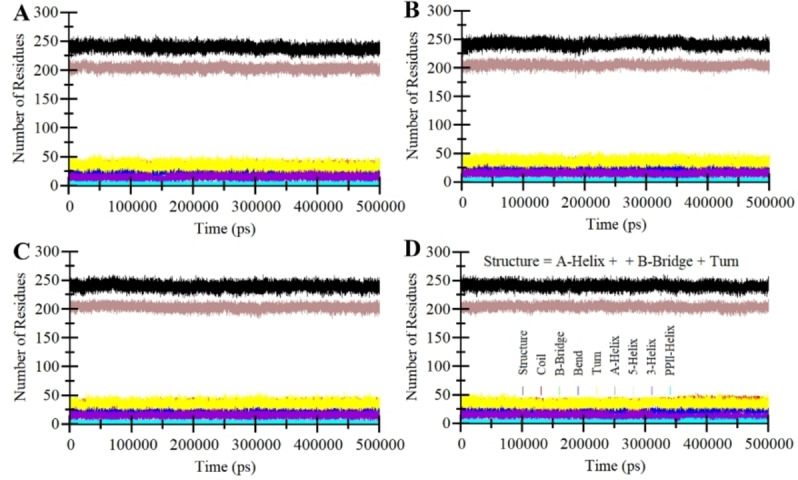
Secondary structural dynamics in free PDE5, PDE5‐Dutasteride, PDE5‐Spironolactone, and PDE5‐Sildenafil.

A protein‘s structural stability and functional characteristics are intricately associated with its secondary structural elements, which dictate its three‐dimensional configuration and the extent of flexibility or rigidity within the system.^[35]^ This study aimed to unravel the complexities of how Dutasteride and Spironolactone interact with PDE5, specifically concerning the dynamics of these secondary structural components. By closely examining the trajectory of these secondary structural elements, we gained a comprehensive understanding of each residue‘s role in shaping secondary structures over time. The graphical representations resulting from this analysis provide a visual depiction of the intricate interplay within the secondary structure (Figure 6). These graphs, illustrating the evolution of secondary structure formation throughout the experiments, collectively illuminate how the binding of the compounds influences the dynamic equilibrium within PDE5. Figure 6A reveals the notable stability inherent in the secondary structural components of PDE5. The secondary structure panels of each system exhibit a consistent pattern over the 500 ns simulation. Importantly, these results emphasize that the secondary structural elements of PDE5 remain largely unchanged throughout the simulation period, whether Dutasteride, Spironolactone, and Sildenafil have interacted with the protein (Figure 6B–D). This observation underscores the robustness of these secondary structural components in preserving the overall architecture of the protein.

#### Principal Component Analysis

**Figure 7:**
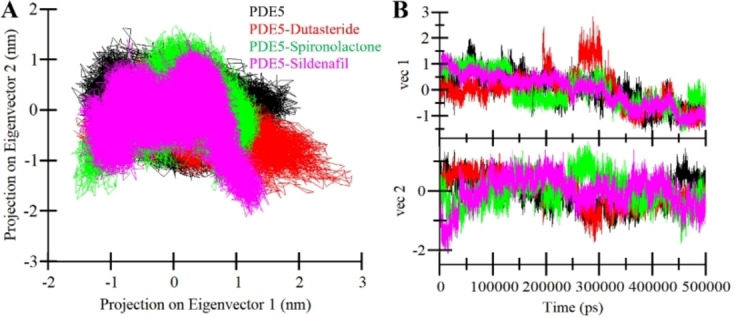
Principal component analysis. (**A**) 2D projection of PDE5 free (black), PDE5‐Dutasteride (red), PDE5‐Spironolactone (green), and PDE5‐Sildenafil (magenta) complex calculated after 500 ns of MD trajectories. (**B**) The time evolution of the projection trajectories.

PCA emerges as a potent methodology for unraveling the collective movements of C_α_ atoms within a protein, as elegantly encapsulated by the Eigenvalues (EVs) derived from the covariance matrix.^[29]^ This technique offers insight into a protein‘s overall dynamic motions and the spectrum of conformational changes it undergoes. Remarkably, PCA′s applicability extends to exploring the kinetics of protein folding, even in the presence of the ligands. In this study, PCA was employed to illuminate the intricate tapestry of conformational motions within PDE5 across all simulated trajectories, encompassing apo PDE5, PDE5‐Dutasteride, PDE5‐Spironolactone, and PDE5‐Sildenafil (Figure 7). In this pursuit, the C_α_ atoms of PDE5 played a pivotal role in calculating the average magnitude of protein motions. The outcome of this analysis unveiled intriguing trends. Notably, the PDE5 complex with Dutasteride displayed notably larger positive movements on EV1 compared to the free PDE5 and its other complexes (Figure 7A). These findings are visually captured through the projected conformations along the first two Eigenvalues, EV1 and EV2. Conversely, the analysis indicated that PDE5‐Spironolactone exhibited relatively more stable behavior, closely mirroring the motions of apo PDE5 while introducing some positive movements on EV2. Figure 7 visually portrays these insights, revealing that the PDE5‐Dutasteride and PDE5‐Sildenafil complexes occupied a broader phase space in the projections compared to apo PDE5 and PDE5‐Spironolactone. All complexes share mostly common phase space before and after ligand binding. This observation carries implications for the stability of these systems. Notably, the robust collective motions within the PDE5‐ligand complexes provide a rationale for the enhanced stability of the docked complexes.

#### Free Energy Landscape Analysis

**Figure 8:**
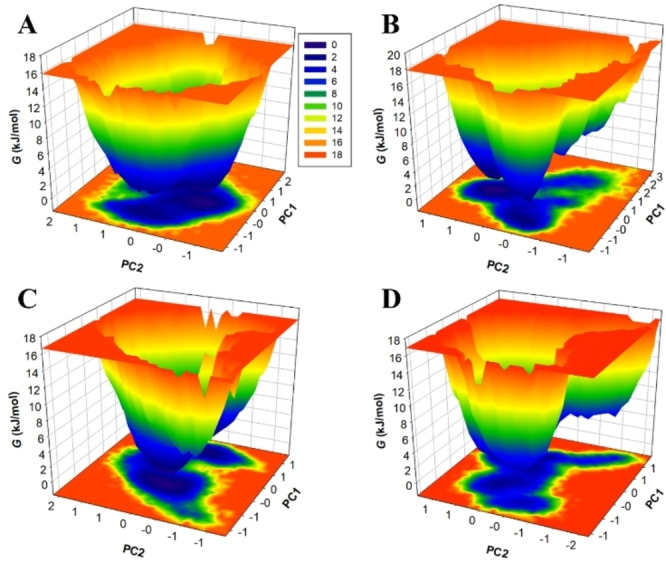
The free energy landscapes for (A) free PDE5 (B) PDE5‐Dutasteride, (C) PDE5‐Spironolactone, and (B) PDE5‐Sildenafil.

The insights held within the first two PCs, i. e., PC1 and PC2, were utilized to explore the intricate conformational dynamics governing PDE5, thus enhancing our understanding of its behavior. This insight is visually represented in Figure 8, where the FELs of PDE5 are graphically depicted, along with its complexes with Dutasteride, Spironolactone, and Sildenafil. In examining the FELs, it′s noteworthy that regions of structural configurations characterized by lower energy levels are depicted in deeper blue, providing a clear visual representation of the conformational landscape. Analyzing the FELs, PDE5 in its unbound state, exhibits a single global minimum confined within a large basin, reflecting its inherent conformational stability. However, the introduction of Dutasteride, Spironolactone, and Sildenafil triggers a significant shift in PDE5′s conformational stability (Figure 8B–D). Contrary to converging towards multiple stable global minima, PDE5′s behavior changes when interacting with Dutasteride, Spironolactone, and Sildenafil, leading to distinct structural transitions but achieving overall global minima confined within 1–2 adjacent basins. In summary, the analysis of essential dynamics reveals that the structural stability of PDE5 remains largely unaffected upon binding with the compounds. This observation underscores the robust stability of the complexes formed between PDE5 and the selected compounds. Overall, the binding potential, biological activities, and stability of Dutasteride and Spironolactone with PDE5 suggest their promising prospects as PDE5 inhibitors. These findings indicate their suitability for further exploration in drug development, with the aim of harnessing their therapeutic potential.

## Conclusions

A detailed investigation of the interactions between FDA‐approved compounds and PDE5 was conducted using a combination of molecular docking, MD simulations, and bioinformatics analyses. The primary goal was to identify potential drug candidates that exhibit strong binding affinity to PDE5, laying the foundation for novel therapeutic interventions. Initially, we systematically ranked a diverse set of FDA‐approved compounds through molecular docking‐based virtual screening to identify those with the highest binding potential toward PDE5′s active site. The elucidated hit molecules were further evaluated based on their drug profiling and PASS predictions for their biological activities. These analyses highlighted two drug molecules, Dutasteride and Spironolactone, as potential inhibitors of PDE5. These molecules established significant interactions, such as hydrogen bonding, π‐π stacking, and hydrophobic interactions, with key amino acid residues of the PDE5 binding site. The MD simulations confirmed the stability and integrity of PDE5‐ligand complexes over extended time scales. Dutasteride and Spironolactone exhibited consistent stability, stabilizing PDE5′s structural dynamics and secondary structure elements. Intriguingly, PCA highlighted the distinct conformational behaviors induced by ligand binding, with Dutasteride and Spironolactone exhibiting unique impacts on PDE5′s dynamics. Taken together, the remarkable binding strengths, versatile pharmacological attributes, and structural insights presented by Dutasteride and Spironolactone establish them as promising candidates for further exploration in drug repurposing against PDE5‐associated disease management. The outcomes of this study contribute to the broader field of drug discovery and development and pave the way for future investigations to harness the potential of Dutasteride and Spironolactone as effective therapeutic agents.

## Conflict of interests

The authors declare no conflict of interest.

1

## Data Availability

All the data is presented in the manuscript.
